# Paper Read before the New Orleans Academy of Sciences

**Published:** 1859-04

**Authors:** John S. Clark

**Affiliations:** Fellow of the Academy, and published in this Journal by permission of the Academy.


### ARTICLE II.

*Paper read before the New Orleans Academy of Sciences.* By JOHN S. CLARK, D. D. S., Fellow of the Academy, and published in this Journal by permission of the Academy.

MAN, from the remotest ages of which history has left any record, has ever been, to some extent, the earnest explorer in the mysteries of creation.

He has seen unfolding the ever changing panorama of physical organization always obedient to perfect law, and has seldom failed to recognize a great first cause as the perfector of a perfect design. This discovery has not been made because man is an observing animal. It is not because he has the power of noting, remembering, or even recording phenomena. But it is because he possesses an interpreter which we call mind or intellect.

Mind we understand to be a scintillation of that divine genius possessed only in perfection by the Creator.

The works of creation, the world we inhabit, and the teeming myriads of worlds, even down to the smallest particles of matter, we understand to be the exponents of a design perfect in organization and movement because they bear the imprint and impregnation of a Divine constructor, a part of his perfection pervading the whole as an inherent impetus. The ontological proof of the existence of a Supreme Being, or that species of evidence drawn from the nature of things, showing that there must be an intelligent first cause sustaining certain necessary relations to the things created, is supposed to offer a sufficient basis for quoting, without apology, the coincident assertions found in what is termed christian philosophy. Need we go behind simple faith in the statement, that "God breathed into his nostrils the breath of life and man became a living soul?"

There must be a point where finite reason staggers and faith takes hold on facts it cannot fathom, founded on principles it cannot elucidate. What finite mind can comprehend infinite time? But before us stretches away the impenetrable veil of eternity. Behind us we totter on the utmost verge of thought to behold old worlds rolling up from an infinite lapse of time. The mind can but grasp one link of the endless chain of time and call that the beginning.

We must put faith in the pre-existent eternity of matter or in an eternal pre-existent creator.

Shall we claim kin with the mere lump of senseless matter ushered into being by the uncaused power of uncreated progressive dust, or shall we recognize the divinity that stirs within us as an emanation from an intelligent creator who works by a perfect design?

True philosophy holds no man shivering on the brink of reason's verge, chained to the task of penetrating the mysteries of eternal distance; she calls for no such far off explorations, but teaches by every muscle and fibre of our bodies which are strung with visible, tangible mysteries, the simple lesson of faith in facts we cannot fathom, in effects whose cause lies as much beyond our solution as the problem of eternity.

Can any philosophy reach farther or discern more in regard to man's intellectuality than that which tells how he became a "living soul?"

In regard to the other proposition, ontology whose proofs are drawn from an examination of the "essential qualities of things," "their nature, possibility, reality, necessity, and mutual operation" warrant the assertion that matter is impregnated with a Divine impetus.

It is a well recognized law in physics that no force or impetus is wholly reproductive.

Power, so far as we know, is an expenditure.

That power that created and arranged the particles of matter, giving quality, position and movement; that power that wheeled the worlds into their orbital paths; that gave force and velocity to the vast imponderable masses; that gave light and heat, must still exist as an active principle, either inherent or constantly renewed, and the faith that embraces the fact of a Creator needs no additions to accept the evident philosophy of continued force. The discovery of a Creator, and a recognition of his hand in his works, is, by the simple principle of affinity, so fixed in the human mind that no man is able entirely to obliterate it.

That man does not exist whose misuse or abuse of intellct amounts to a full soul-belief in the non-existence of a Creator.

As well might you amputate a sunbeam for light as to use that of a living soul to deny its paternity.

Whatever may have been the few isolated attempts of some to deny it, man accepts the solution of his endowment from the hand of a Creator, and he walks forth among the mysteries of the material world as an explorer bearing the key to as much as his finite powers can grasp.

Matter in every form we find subject to certain fixed laws; to ascertain them is the legitimate work of the scientific explorer.

It is not the accumulation or compilation of them into a mere mass of recorded facts, for men may gaze and watch and note the changes in nature until they outstare the blinking stars.

They may witness phenomena, record experiences, and enact pyramidical monuments of industry, but never rise higher in science than the "hewers and the drawers of water," for it is only when the intellectual interpreter rises from the facts to the philosophy, and by its comprehensive view of the unity of law that pervades the whole and gives the partial solution that any investigation can be said to assume the dignity of a science.

The eye may be opened on a single object and exhaust itself in the single view, but it is only when it receives the measured lines of hill and dale, lights and shadows that the landscape is mirrored there.

So that the intellectual spark may glimmer mid the upheaved masses of an Etna or Vesuvius or even wake dull reflections from the beds of "old red sandstone," but it never becomes to man the delineator until it traces out those lines of living light as they converge into one system as parts of our stupendous whole, whose beauty is in their unity, whose laws are traces of infinite omnipotent perfection.

These laws are also discovered by affinity.

What is it that steals over the senses, giving intense delight as we gaze on some of the higher works of art? Is it because a Raphael or a Michael Angelo have placed before us something new or original? something more beautiful than can be found in nature? On the contrary, the artist's merit lies solely in his close imitation of nature. It is that by affinity we recognize the path of mind, and entering it, a kindred spirit bears us company, and with him we revel in the sublime delineations of his thought pencil.

It is not the marble or the canvass, with their blended hues and proportions that so delight us, nor even the mere pleasure of being brought as it were face to face with the model subject, but it is to the touch of genius that we yield to the rapture of kindred intellectualities.

Thus in nature it is not the mere shape, form or feature, it is not the blending of lights and shadows as we gaze on her open face, but it is that the great artist has placed there the symbols of his own infinite genius, and our finite but God-lent moiety of the same power recognizes as it were a part of its own essence, and the landscape glows as with a living exhalation.

To the mere organ of sight the highest mountain pile is but a cold fact, the ocean rolls up but a succession of monotonous waves, but to the mind they speak of vastness and sublimity.

We enter then on the study of nature or physics with a prestige that points out the path of investigation.

That glorious path, trodden by all the philosophers from the earliest ages, illumined as was that of the shepherds of old with more than a "cloud by day and a pillar of fire by night."

What a glowing history would that not be which should lead us back to those ages in whose glimmering twilight there flashed out, here and there, a star whose struggling rays lighted the first altars to science.

With what interest would we visit the secret laboratory of the recluse, watch the enchanted wanderers o'er the hills of Greece, listen to the savans and scholastic representations of Aristotle and Gallileo until we behold those beacon fires of nations, those way-marks of the world that shone forth from the hand of a Locke, a Bacon, and a Newton.

But enchanting as that history is, even with the imperfect records they have left on its pages, we are compelled to turn to that which if it lies not deeper, extends more broadly over the surface of man's exploits in the use of intellectual power.

We are informed that the very first use of that power was to "aspire to be gods."

Whatever may be the confidence placed in the Mosaic history, there are some things so self-evident that we feel under no apprehension of mistake as to their verity. This simple statement is so perfectly consistent with, and explanatory of, man's humanity, as shown on every page of his history, that it comes to us with all the force of a self-evident truth.

That men aspiring to be gods, and failing in power, should attempt miserable imitations with all the shades of pretence and assumption of what they are not, accords with our experience.

In accordance with this we find him. first seeking the means of subjugation under false pretences.

If God governed some of the primitive races by a theocracy with human and superhuman agencies, man has covered the pages of history from the earliest ages down to the present, with one continued recital of conquests between rivals claiming authority, either as delegated by some omnipotent power, or pretending to possess in themselves, that of gods.

Add to this the fact, that every swindler is a thief, at heart, and you have ample proof of the statement of that much abused historian, whose writings are as yet about as far from being understood, as the just dawning interpretations on the exhumed tablets from the buried treasures of the Nile. In the world's conquests, the enlistments are generally under the banner of treason, to man's legitimate inheritance. Men in a contest for great principles, involving human rights, may become invincible. But men, seized upon by this ignoble fanaticism, that borrows power from assumption (swindling,) and the promise of acquisition, (thieving,) become devils. The one, waking from the slumbers of a long night of oppression, rolls back the tide of wrong on tyrants and their thrones, and then lays down the implements of warfare and seeks for peace and happiness.

The other keeps a standing army ever on the alert for conquest. The one occupies here and there a bright page in history, the other covers its darkened pages with blood and crime.

Look back at the dark tide of human misery that has rolled down the stream of time with its crushed and bleeding victims. Whence come they with their centennial accumulation ?

Are they the discomfited opposers of some great fundamental principle ? Are they not the world-marked monuments of man's assumption ?

It must be apparent, that a sound philosophy in regard to human rights is necessary to the high position man ought to assume in the scale of being, for its principles are but laws from the Great Law-giver.

So science, in all forms, being but traces of divine law, must ever be the friend and conservator of our highest elevation in our moral, social and physical relations. She has ever answered the call of man's necessities.

Does he faint by the wayside for want of strength, she lends him the arm of a Hercules.

Does his eye become dim, as he studies the heavenly bodies? She brings as it were down from their ethereal pathway the planets as they roll and passes them across the narrow boundaries of his vision. Do the infinitesimal motes that float in the sunbeam baffle the natural capacities of that organ? Through the microscope she opens another world replete with wonder and beauty, giving form, shape and symmetry to the atomic infusions of earth, air and water. Is he sick? Her gentle footfall can be heard wherever suffering humanity utters the language of pain. Is the yearning sympathy of friend for friend too slow, as it rides on the wings of the wind? She speaks it to the lightning flash and continents of space are annihilated.

Does he look forward to the hour when this chrysalis investiture shall fall and mingle with its native dust?

She points to that land where science is literally "to know," and philosophy discovers her perfect law.

This, however, is a view of science, in its full unobstructed and legitimate application to our wants and necessities. But those rays of light, calculated to ameliorate our condition, and fall like sunlight on our pathway, are not all destined to fulfil their benevolent mission.

So far as the laws regulating matter are combined and their forces applied directly in purely demonstrative specialties, such as mechanical forces and chemical affinities, there is no obstruction.

But when powers equally, but more occult, requiring man's judgment and veracity, as a constantly recombining medium, such as the application of science to the healing of moral, mental or physical ills, there intervenes the full power again of assumption, and instead of the good Samaritan, there stands forth the empiric, claiming again the skill-power of Omnipotence.

So true is the poet's description of those who steal the livery of the court of heaven to serve the devil in, that there is not a garb that science, as the angel of charity wears, that is not stolen for the flaunting vesture of armies of ignoble pretenders and pseudo-scientific missionaries.

There is not a pure heart-throbbing sympathy that illumes the very shadow of a Florence Nightingale, as it falls on the poor soldier, wounded in the battle of life, that is not daguerreotyped for the drapery of a babbling crowd of mock philanthropists.

It may be thought an easy task to detect the spurious imitations of science, and scientific men may laugh at the follies of the deceived, but all are not men of science.

"Primary, secondary, and tertiary formations," mark no periods in the world's age to the common eye. It reads no history there, but, to the geologist, they roll up the curtain of the past, and speak of periods beyond man's proudest monuments. The great mass are compelled to stand at the outer gate of the temple, and when strange fires illumine the outer courts, who shall open the portal to her inner light, that her modest rays may dissipate that false zone that dazzles the sight by its meteoric glare. It so happens that in those very sciences that concern us most as matters of practical importance, the exhibition of intellectual power on the side of fraud, is of no mean type, and it seems as though the assiduity, subtlety, acuteness, and mental vigor, is increased just in proportion to the magnitude of the fraud to be perpetrated.

We look with wonder upon the adroitness of the pickpocket and skillful gambler. We almost respect the talents of the ingenious thief, who, under the very eye of vigilance, cuts into our strong box and carries off our gold, and we admire the genius of the successful counterfeiter of our bank notes, and wonder why those men develop resources of such amazing skill, when honest men find it so hard to accomplish easier feats.

But they are not isolated cases of rare development, but mere shades of that natural tendency to unusual vigor, when men assume false positions in moral or mental acts.

Take a boy without education; tell him that two and two make four, urge him through a three weeks' study to learn the multiplication table, and pronounce him a dull boy.

Now, give that boy a pack of cards, and he will master a game of brag in fifteen minutes.

Place before him history, and he will yawn over its glowing pages, and fill his mind with a strange juxta-position of kings, knights, warriors, kingdoms and continents. If you require him to write a composition, he labors like an Ajax, tugging away at his unaccommodating brains until he whines out his despair of ever catching an idea.

Give that boy a copy of Sinbad the Sailor, or the Arabian Knights, and he will burn his first midnight oil over their contents, and in twenty-four hours will write you brilliant tales of the fairy land, with clever imitations of their impossible achievements. When this young man has attained to about half of a very meagre education—when the principles of mental philosophy are to him a vast monument of unmeaning logic, and Butler's Analogy, even too stale for his delicate understanding. Send him forth into the wide, wide world, and let him meet there one of those modern specimens of phsycological, biological or spiritual professors, who, in one hour, will break down that barrier of reason which separates the real from the unreal, and you will behold one of the strangest transformations in nature. You may expect to laugh at his mistakes, but give him three weeks, and you will open your eyes in amazement at his intellectual strides. He will toss systems of philosophy about as adroitly as the juggler does his balls, and set others afloat as easily as a child blows bubbles in the air. Suppose you attempt to follow him in his mental flights. He sails away, skimming the entire circle of the "Sciences," which he treats as familiar, but exploded dogmas, and perching himself on some ethereal eminence, regales you with the music of the spheres; sends down samples of poetry, fresh from the pen of Milton, Sancho-panzaisms from Cervantes, and even violates the sanctity of our "patriot graves," for endorsers to his unrighteous frauds.

This sudden mushroom growth on the face of society, even down to the infusoria that floats on its troubled waters sending forth its malarious breath, is certainly as worthy the attention of the students or professors of science, as that which comes up on the earth's surface in a night, or floats on our waters under a summer sun. It springs up as naturally from the rank soil of the human heart. Even an indolent boy, who drags his slow length along to school and idles away his time when there, will fill up every moment of truant hours with the most active employment.

Let an indolent man, one who hates locomotion, steal an article of value, in some crowded thoroughfare, and being detected, hear the cry of "stop thief," and his inactivity will vanish in good specimens of tall walking. So let a man break over the barriers of truth and reason in his mental acts, and all nature utters the cry of "stop thief," in his ear, and he hurries on from one falsity to another with amazing celerity.

The skulking thief, that flits like a twilight shadow across your path at night, and the bold brigand that sweeps the plain like the wind and hies away to his mountain home, are but surpassed in activity by a motley crew of petty swindlers, traffickers in human credulity. Patent venders of omnipotent powers and skill. Fanatics of all grades up to the raving maniac, all are active. We have no need to seize the telescopic glass of the theologian to range the fields of the spirit land for armies of fallen angels that are said to infest that country, for we have him in active contact with us, an army of moral and intellectual corsairs, ever vigilant, ever active, who sweep over the face of society like the sirocco of the desert, to whose fiery breath, the nations bow down in silence.

Gentlemen of the Academy of Sciences, and all who form a part of the advance army of explorers, when these prostrate sons of men look to you, and ask for bread, will you give them a stone?

As conservators of the high principles of a high philosophy, will you withhold the glimpse of her perfect circle, as drawn by the hand of Omnipotence? They ask to look into *your mirror* for traces of nature's *open face.* *Will you, dare* you, substitute a miserable daguerreotype of your own?

